# Advances in Artificial Intelligence (AI) Models and Generative Algorithms Represent a New Paradigm for Genomics Research

**DOI:** 10.3390/ijms262210925

**Published:** 2025-11-11

**Authors:** Du Hyeong Lee, Eun Gyung Park, Yun Ju Lee, Hyeon-su Jeong, Hyun-Young Roh, Ga-ram Jeong, Sang-Woo Kim, Heui-Soo Kim

**Affiliations:** 1 Department of Integrated Biological Science, Pusan National University, Busan 46241, Republic of Korea; doo2080@naver.com (D.H.L.); ehdtodt@pusan.ac.kr (E.G.P.); lsg5821@naver.com (Y.J.L.); tbd97@pusan.ac.kr (H.-s.J.); susan9416@naver.com (H.-Y.R.); gracefka@naver.com (G.-r.J.); 2 Institute of Systems Biology, Pusan National University, Busan 46241, Republic of Korea; kimsw@pusan.ac.kr; 3 Department of Biological Sciences, College of Natural Sciences, Pusan National University, Busan 46241, Republic of Korea

**Keywords:** machine learning, artificial intelligence, generative algorithms, genomics, deep learning, bioinformatics

## Abstract

Genomics has developed in step with progress in computing. As computational capabilities have grown, analyses have expanded from simple statistics to artificial intelligence (AI)-based approaches within genomics. The decline in sequencing costs has led to the accumulation of diverse genomic datasets, rapidly accelerating AI for genomic analysis. AI models are now developed and applied across many functional domains, including the prediction of transcription factor binding sites, epigenetic elements, DNA methylation, and noncoding sequence functional annotation. With the maturation of architectures such as deep neural networks, convolutional neural networks, recurrent neural networks, and transformers, many genomic models now accommodate longer inputs, capture long-range context, and integrate complex multi-omics data, thereby steadily improving predictive accuracy. Moreover, the emergence of generative AI has enabled models that can simulate and design genomic sequences. The introduction of generative AI into genomics goes beyond inferring function to the capability of replicating functional genomes. These advances will help advance genome interpretation and accelerate our ability to chart and navigate the genomic landscape.

## 1. Introduction

**Figure 1:**
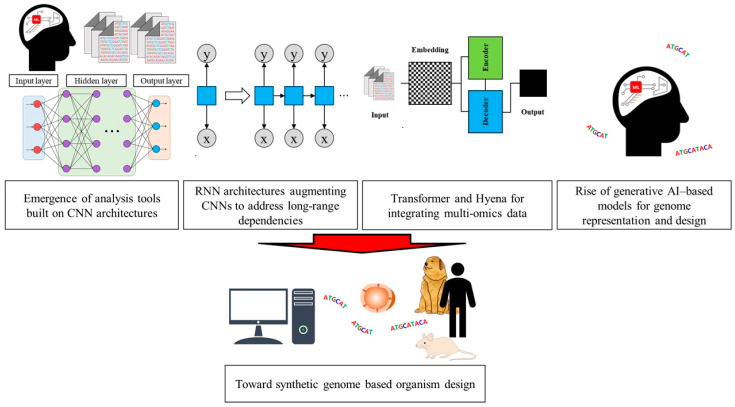
Schematic illustration of the development of genomics and the advancement of AI-based genomics research.

The rapid development of artificial intelligence (AI) is transforming life for people in a variety of different fields. AI models quickly augment traditional search engines, tools for data exploration, drafting, and administrative assistance [1,2,3]. This paradigm involves the replacement of traditional statistical analysis tools with faster and more efficient mechanisms not only in daily life but also in many areas of science, especially biology. AI is being used in a variety of fields of biology, such as determining the prognosis of cancer patients, developing new diagnostic tools, and predicting the outbreak of infectious diseases [4,5,6]. Current AI is performing roles ranging from simple data classification to generalizing patterns and extracting key information from large datasets generated by high-throughput molecular technologies [7]. Traditionally, genome analysis has included some types of tasks such as classifying short sequencing reads, using GWAS to identify polymorphisms associated with phenotypic traits and diseases, and applying eQTL analysis to pinpoint genomic loci that influence gene-expression levels [8,9,10]. However, advances in computing and machine learning have spurred a growing body of work that uses deep-learning algorithms, such as artificial neural networks (ANNs), to infer phenotype-associated genes and genomic variants, predict protein functions, and model the structure of the genome [11]. In particular, with the development of the transformer architecture and self-attention–based systems exemplified by AlphaFold in analyzing protein structures, AI has been used to predict protein structures using large-scale data and for whole-genome research since the 2020s, and the use of AI in genome research is increasing [12,13]. Since then, AI models have been employed in the field of pharmaceutical research, including the development of treatments and vaccines for the novel coronavirus (SARS-CoV-2), and the use of AI in biology has seen a further increase [14]. The 2024 Nobel Prize in Chemistry was awarded to David Baker, Demis Hassabis, and John M. Jumper for developing AlphaFold 2, thus further raising awareness of AI in the context of biological research [15]. AI models now classify genomic data to infer disease risk and predict structure; they also synthesize novel gene or genome sequences conditioned on user prompts [16]. In this review, we will examine the advancement of genomics research, the use of AI, and genomics research using generative AI (Figure 1).

## 2. Machine Learning and Deep Learning Algorithms Used in Genomics Research

With developments in next-generation sequencing technologies driving down sequencing costs, genomic data have accumulated across diverse domains. Vast resources have been assembled, including consensus sequences from multiple organisms, genomes of newly characterized species, ChIP-seq/ATAC-seq datasets that capture interactions among regulatory factors, and CLIP-seq profiling protein–RNA binding [17,18]. As these datasets have grown, GWAS have been widely conducted to explore relationships between genetic and genomic variation and phenotype and to analyze genomic function [9]. In parallel, advances in bioinformatics tools have made computing-centric analyses, from detecting SNPs associated with disease to predicting alternative DNA conformations in silico [19,20]. During this period, early machine learning algorithms emerged. These approaches, grounded in statistical modeling to maximize likelihood or in similarity-based rules for binary classification, include logistic regression, random forests (RF), boosting methods, k-nearest neighbors (k-NN), support vector machines (SVM), and Naïve Bayes (NB) [21,22,23,24,25,26]. Such models have been applied in genomics to infer SNP function and elucidate genotype–phenotype links, supporting applications such as cultivar development and the discovery of disease-causing variants (Table 1) [27,28,29].

With developments in computing, ANNs began to be applied in earnest. ANNs are models that solve problems by adjusting connection weights among multilayer nodes, an abstraction inspired by biological neural networks [11,43]. Although early performance did not clearly surpass techniques such as SVMs or NB due to limited computational resources, improvements in hardware and the accumulation of data led to the rapid spread of deep neural architectures such as deep neural network (DNN)/multi-layer perceptron (MLP), convolutional neural network (CNN), and recurrent neural network (RNN)/long short-term memory (LSTM) in genomics after 2015 [44,45,46]. Furthermore, the advent of the transformer architecture catalyzed large-scale natural language models, and self-attention–based systems exemplified by AlphaFold, ushering in a new paradigm after 2020 in which AI research increasingly integrates diverse, large-scale datasets [12,47]. As a result, the accumulation of massive genomic data and dramatic gains in computational power accelerated the adoption of AI in genomics and drove substantial innovations in genomic function analysis.

### 2.1. Learning Paradigms and Characteristics of Deep Learning Models Used in Genomic Research

Recently, deep learning-based models used in genomics are categorized by learning paradigm into supervised, unsupervised, and semi-supervised learning. In supervised learning, genomic data are accompanied by labels or annotations such as transcription start sites, transcription termination sites and splice sites. In contrast, unsupervised learning discovers latent patterns from large, unlabeled datasets [48,49,50]. In genomics, supervised learning trains predictors using known biological annotations, while unsupervised learning can be applied to uncover the structure of extensive variant and sequence data [51]. Supervised learning underlies many deep learning-based early genomic analysis models such as DeepBind. It often achieves strong predictive performance and is well-suited to fine-tuning of pretrained models. Still, it can be limited by the difficulty of data collection and the risk of overfitting. By contrast, unsupervised learning is easier to scale in terms of data acquisition and provides a foundation for pretrained models such as DNABERT, yet it carries the risk of learning spurious patterns [52,53,54]. Across these learning processes, the most critical steps are data acquisition and preprocessing, such as normalization and length handling. As dataset size increases, the risks of overfitting and underfitting generally diminish, and appropriate preprocessing can substantially reduce computational cost [54]. Selecting an architecture that matches the analysis objective and input data characteristics is essential, as each deep learning model entails its own advantages and disadvantages.

### 2.2. Characteristics of Major Deep Learning Architectures Used in Genomic Research

DNN and MLP apply successive nonlinear transformations to vector inputs through multiple hidden layers. They are fast and relatively easy to implement and modify. They can ingest heterogeneous input types and are straightforward to integrate with other deep-learning architectures [44,55]. Using at least 2–3 hidden layers is recommended, and employing more than 100 hidden layers can be effective. Compared with traditional machine-learning models such as NB, k-NN, RF, and SVM, DNNs often achieve superior accuracy. However, because all neurons are fully connected, the number of parameters grows combinatorially with input dimensionality, as in high-dimensional genomic data, so achieving reasonable performance can require substantial computation time [56,57]. Consequently, in genomics, standalone DNNs are more often used in auxiliary roles rather than ingesting large raw sequences directly, for example, by taking functional/disease-association features such as GO, PPI, PathDIP, KEGG as inputs to predict aging-related genes, or by learning short (<100 bp) sequence windows using enhancer-related histone-modification signals as in EP-DNN [58,59]. In short, while DNNs are simple to build and can outperform conventional statistical models in accuracy, they offer limited parameter efficiency and, due to their fully connected design, are not resource efficient.

CNNs replace the fully connected structure of DNNs with convolutional layers that exploit local patterns and weight sharing, yielding high parameter efficiency and fast training relative to model size [60,61]. However, CNNs typically require fixed-length inputs, which can introduce information loss and, for large contexts, may entail longer training times [62]. In genomics, CNNs have been used for tasks such as predicting the binding specificities of DNA/RNA–binding proteins in DeepBind and DeeperBind, and annotating functions of noncoding DNA regions in Basset and DanQ [53,63,64,65]. As deep learning methods have been applied to genomics, many supervised models have been built on CNNs. Owing to their relatively low implementation complexity and strong accuracy, CNN-based approaches remain widely used. That said, to address long training times in long sequences and limitations in modeling long-range dependencies, CNNs are now often combined with other architectures.

RNNs and their variant LSTM can model long-range dependencies more precisely than CNNs, and have thus been used to predict interactions between distantly spaced nucleotides. Their strength with variable-length inputs makes them well-suited to genomic data [66]. RNNs have been employed in model DeepZ to predict Z-DNA structure, and LSTMs have been employed in models such as AttentiveChrome to predict chromatin interactions [67,68]. Because RNNs handle larger inputs and long-range dependencies more effectively than CNNs, many CNN-based models have been augmented with recurrent components.

Transformer architectures learn long-range interactions and global context effectively via self-attention and, as with BERT, a natural language processing model, are well suited to parallelization and to pretraining and transfer learning. Because they integrate heterogeneous data readily, many AI models developed since 2020 have adopted transformer architectures [69,70]. In genomics, supervised models such as Enformer target enhancer-associated prediction, while pretrained approaches like DNABERT, using k-mer tokenization, are widely used. These models enable the integration of diverse omics signals, including gene-expression regulation, transcription-factor binding sites, and chromatin accessibility [54]. With their unique scalability and appropriate fine-tuning, DNABERT can infer a variety of biological factors, including chromatin marks, transcription factor binding domains, and genome functions. However, transformers typically require substantially more training data and computational resources than other models [71]. Consequently, many studies extensively fine-tune pretrained models such as DNABERT to obtain task-optimized analyzers.

Hyena is an architecture designed to relax the transformer’s relatively short effective context length, efficiently handling ultra-long-range context while maintaining single-nucleotide resolution. It has the potential to achieve high performance with comparatively few parameters, but it can be challenging to fine-tune [72]. Hyena has been applied to the genome-generative model EVO, where it demonstrated strong performance [16]. Although Hyena can accept very large inputs and thereby capture genomic characteristics well, it still requires extensive validation. Models currently used for genome analysis are being developed using various deep learning algorithms. The development trend is evolving toward building complex models by integrating large amounts of data and omics data over time (Table 2).

## 3. Deep Learning Models Being Developed and Utilized in Various Fields of Genomic Research

Since deep learning demonstrated its potential in biology, a wide array of models has been developed and applied in genomics, centered on predicting the functional consequences of sequence variation. In particular, AI is actively developed and leveraged in areas that are difficult to resolve solely by experimentation or by existing annotated reference resources, including sequence-based prediction of transcription factor binding sites, regulation of gene expression (promoters, enhancers), epigenetic marks, alternative splicing sites, functions of noncoding RNAs, and detection of alternative DNA conformations.

### 3.1. Prediction of Binding Regions Between Nucleotides and Proteins

Predicting the binding regions of proteins such as transcription factors or regulatory factors are central to understanding gene expression, translation, and alternative splicing in genomics. DeepBind was the first to apply a deep learning approach in this prediction, training CNNs on data such as ChIP-seq and CLIP-seq to estimate binding propensity. And Basset used RNA-seq data to learn accessibility-based features to predict transcription factor binding potential in noncoding regions [53,64]. Likewise, the CNN-based BPNet inferred transcription factor binding motifs and captured them at base-level resolution, improving accuracy [73]. Afterwards, to address CNNs’ limited ability to model long-range dependencies, hybrid architectures that merge CNNs with RNN/LSTM layers were developed DeeperBind, DanQ, and iDeepS to predict DNA/RNA–binding protein interaction sites more accurately [63,65,74]. Although these CNN–RNN hybrids improved accuracy over CNNs alone, only modest gains were achieved, and the inability to learn very long sequences remained. To overcome these issues and to harness protein language models like ProTrans together with attention mechanisms, the transformer-based TransBind was proposed, achieving 97.68% accuracy and surpassing previous CNN and CNN–RNN models in binding-site prediction [75]. These advances in deep learning models can aid the discovery of previously unknown transcription factor binding regions, deepen understanding of gene-regulatory mechanisms, and help prioritize candidate regions efficiently during pre-experimental design.

### 3.2. Prediction of Expression Regulatory Regions and Estimation of Epigenome Characteristics

The importance of noncoding DNA regions that do not encode proteins has grown substantially in the regulation of gene expression. Although many analyses in genomics aim to elucidate the functions of noncoding DNA, predicting how noncoding regions influence gene expression across diverse tissues and cell types remains challenging. Gene expression can be affected by the sequence features and epigenomic states of noncoding regions [76]. DeepChrome trains a CNN on labeled histone-modification marks within 10 kb around the TSS to predict gene expression strength. Compared with traditional ML approaches, DeepChrome achieves a higher AUC of 0.80, versus 0.66 for SVM and 0.59 for RF [77]. Subsequently, AttentiveChrome employed LSTMs to more precisely capture regulatory context [68]. In addition, because DNA methylation affects gene expression yet has often been assessed primarily by experimental means, CNN-based predictors such as DeepCpG and CpGenie were proposed to infer methylation status directly from sequence [78,79]. Building on these advances, Enformer has recently been developed as an integrated tool that simultaneously predicts gene expression and epigenomic features. Applying a transformer-based architecture, Enformer integrates distal regulatory information up to 100 kb away to model enhancer–gene links and predict expression [19]. Enformer outperforms prior approaches and, owing to its extensibility, has served as a foundation for newer genome-function prediction models such as Borzoi [80]. However, because Enformer is trained primarily on genome sequence–level labels, its sequence-function prediction can be somewhat limited when generalizing to novel cellular contexts. Addressing this, EpiGePT, which integrates transcription-factor RNA-seq and 3D chromatin-contact information, shows superior sequence-function prediction compared with Enformer [81]. Overall, the latest regulatory region prediction models tend to integrate large-scale context and multi-omics data to further enhance predictive power.

### 3.3. Splicing Prediction

Alternative splicing is one of the key determinants of complexity in eukaryotic transcriptomes. It is implicated in a wide range of biological processes, including species-specific cell differentiation, telomere length maintenance, and diseases such as cancer and autism spectrum disorder. Nevertheless, identifying splicing signals and predicting their activity remains challenging [82,83]. To detect splicing alterations, CNN-based models such as DeepSplice and SpliceRover take splice-junction sequences as input and identify splice variants. DeepSplice achieves 96.1% accuracy, and SpliceRover likewise attains 96% accuracy [84,85]. SpliceAI was a CNN model trained on GENCODE mRNA transcript data that predicted splice junctions, and it identified a significant increase in variants accompanied by splicing alterations in patients with intellectual disability [86]. By detecting splicing changes and enabling base-level tracking of variant-induced alterations, these models hold promise for clinical interpretation.

### 3.4. Pre-miRNA Prediction and miRNA Target Prediction

miRNAs are post-transcriptional regulators that predominantly bind to the 3′UTR of target mRNAs to repress translation. Although tools such as miRDeep2 exist to predict miRNAs from RNA-seq data at the genome scale, their accuracy has been limited, necessitating experimental validation [87]. To address this, CNN-based models emerged deepMir, which takes RNA sequences as input, and miRDNN, which ingests secondary-structure features and minimum free energy (MFE) values [88,89]. Subsequently, to facilitate the use of secondary-structure information and achieve higher accuracy than CNNs alone, the transformer-based miRe2e was proposed [90]. miRe2e accepts raw genomic sequences and processes them through three components (structure prediction, MFE estimation, and a pre-miRNA classifier), achieving higher accuracy than deepMir. Predicting miRNA-target gene interactions is also a major challenge. For miRNA–gene interaction prediction, earlier tools relied on seed complementarity and minimum-free-energy rules, but suffered from high false-positive rates. Although deep learning integration in the miRNA field is comparatively sparse, RNN-based DeepTarget and LSTM-driven approaches such as SG-LSTM-FRAME have been proposed [91,92]. SG-LSTM-FRAME embeds gene–miRNA sequences or topological information and is trained with verified interaction labels from resources like miRTarBase, attaining an AUC of 0.93. This represents a substantial advance for noncoding RNA–gene interaction prediction without direct experimentation.

### 3.5. Prediction of Non-Canonical DNA Structure

DNA commonly has a right-handed (B-DNA) but, under tension stress, can adopt left-handed conformation (Z-DNA). Z-DNA has recently attracted attention as diverse biological functions have been elucidated [93]. Z-DNA was previously predicted using thermodynamic methods, but accuracy was limited, and given their flip-on feature, they are also difficult to verify experimentally [94]. To predict Z-DNA with deep learning, the DeepZ model, which combines RNN and CNN architectures to compute the probability of Z-DNA transitions, has been proposed [20,67,95]. DeepZ assessed the probability of Z-DNA formation and achieved higher accuracy than existing thermodynamic models and nearly identical accuracy to ChIP-seq results. The advent of a more accurate predictive model prior to experimentation can improve the accessibility of Z-DNA research.

### 3.6. Biological Function Prediction in Genomic Sequence

In the early days, genomic sequence analysis tools included methods such as DANN that used DNNs to detect variants associated with diseases or phenotypic changes [96]. Recent models enabled by architectures such as transformers that can handle complex multi-omics inputs are moving beyond single-function analyses to attempt genome-wide functional annotation via pretraining and fine-tuning. A representative example is DNABERT. Through unsupervised pretraining with k-mer tokenization, DNABERT learns upstream and downstream context to capture the global functions of DNA [54]. DNABERT has outperformed state-of-the-art predictors such as BPNet and Enformer in tasks including promoter detection, TF-binding site prediction, motif analysis, splice-site identification, and variant effect prediction. As an unsupervised model, DNABERT can pretrain on unlabeled sequences and then yield broadly useful representations via downstream fine-tuning, making data acquisition more tractable than for Enformer. However, DNABERT, which directly utilizes the BERT architecture, also had limitations. To address transformers’ high data/computation demands and short input-length constraints, the Hyena-based HyenaDNA was proposed [97]. Whereas DNABERT typically accepts inputs of <512 bp, HyenaDNA maintains single-nucleotide resolution for sequences up to 32 kb. Subsequently, another transformer-based model for genome functional analysis, the Nucleotide Transformer, was proposed to improve the limitations of DNABERT. Using unsupervised learning, it can detect genes, introns, coding and noncoding regions, and variants [98]. After fine-tuning, Nucleotide Transformer achieved a higher Matthew’s correlation coefficient than DNABERT, Enformer, and HyenaDNA. Taken together, deep learning models in genomics show a trend of improving accuracy in step with technological advances. However, improved long-range context modeling does not necessarily translate into stronger zero-shot performance [99]. Multi-omics integration and task-specific fine-tuning remain critical. The architectural progression from CNN, RNN, transformer/Hyena, combined with pretraining and multi-omics integration, continues to raise the resolution and accuracy of functional annotation, often down to single-base resolution. However, these gains typically come with increased demands for computational resources and larger training datasets (Table 3).

## 4. Generative Algorithms Are Also Used in Genomics and Genetics to Advance Sequence Design and Data Augmentation

With the onset of the 2020s, AI moved beyond primarily classificational tasks to generative models that synthesize and reconstruct content from learned representations in response to user prompts. These generative algorithms construct images or sentences based on input prompts and conduct further learning based on the input data [100]. More recently, generative algorithms have begun to influence biology and genomics, spurring efforts to reconstruct genomic elements and to recapitulate characteristic genomic features.

Generative algorithms are increasingly aiding the conduct of genomics research. As a representative example, conversational AI like GPT has been used to analyze and categorize genomics research articles, enabling the inference of about 80% of organismal traits and the extraction of roughly 61% of marker–trait associations [101]. Similarly, large language models (LLMs) such as Gemini and Grok are also utilized in fields such as clinical reasoning [102]. Generative adversarial networks (GANs) learn from random noise via generator–discriminator competition to synthesize sequences and images, and they can model and generate characteristic patterns of genomic sequences [103,104]. Generative models such as GANs can synthesize additional training data and are used in genomics to augment datasets by generating synthetic genomes and transcriptomes [105]. There have been attempts to utilize GANs to generate specific DNA sequences and replicate genomic features [106]. As genomic datasets expand, an individual’s gene and whole-genome sequences constitute sensitive personal information; together with increasingly stringent privacy protections, this hampers access to the DNA sequence data required for training. Consequently, the amount of data that can be leveraged for AI training is limited, which may degrade model performance [107]. To address these limitations, researchers have sought to recapitulate genomic features and generate artificial genomes. For example, Burak et al. trained GANs and Boltzmann machines on data from the data of 1000 Genomes Project and approximately 2000 Estonian genomes, producing high-quality artificial genomes that preserve key properties of real genomes. These artificial genome datasets can serve as valuable additional training data for downstream machine-learning studies [108]. Generative models such as GANs have captured genomic structure with considerable precision, opening new avenues for genomics research that directly generate genomes. Nonetheless, important limitations remain; for example, GANs still struggle to read and analyze longer sequences in a single pass.

The genome can be viewed as a collection of nucleotide sequences over a four-letter alphabet. This perspective has motivated efforts to directly apply LLM architectures to genomic data. LLM-based methods are now used across many areas of genomics to infer disease risk, predict the phenotypic effects of genetic variants, and inform personalized diagnostic medicine [109,110,111]. A representative architecture is the transformer, which underpins systems such as ChatGPT. An expanding body of genomic research now leverages transformer-based models. For example, DNABERT trains on large-scale sequence data with deep transformer stacks to predict regulatory elements in the genome, including promoters, splice sites, and transcription factor binding sites [54]. Beyond these examples, transformers have been applied across diverse models for genomic data analysis. In bacteria, transformer-based models identify transcription start sites, translation initiation sites, and DNA methylation loci to provide insight into transcriptional processes, and in human functional genomics, they are used to build models that infer phenotypes directly from DNA sequence [98,112,113,114]. However, in the field of genome generation, transformers require substantial computation and struggle with very long sequences [115]. This means that the basic unit for learning and constructing DNA sequences can be shortened, making it difficult to perfectly reflect the characteristics of the genome, which serves as a catalyst for the need for next-generation architectures. Nguyen et al. present EVO, a truly genome-generating AI. The EVO genome-generating algorithm is based on StripedHyena, considered a next-generation LLM architecture, and addresses the problem of the transformer’s difficulty in processing long information due to its token size [72,116]. EVO is designed to conduct large-scale token unit learning of 2K size, using a multimodal algorithm that predicts multiple factors rather than a single model, such as proteins, regulatory regions, DNA, and RNA, to reconstruct the features of the human genome [116,117]. These technological advancements mark the beginning of a path toward biological design. As technology advances, it becomes harder for humans to understand how AI works. In fact, there are even academic fields dedicated to figuring this out [118,119]. However, these advances offer many new perspectives on the current understanding of organisms in the field of genomics and may even lead to solutions to potential problems in genome design that even humans cannot identify.

Taken together, these perspectives and developments suggest that, in the near future, it may be possible to develop algorithms that generate genomic sequences from phenotypic information or, conversely, predict phenotypes from sequence data. Current genome design using generative AI is limited to how closely it infers existing sequences and how well it represents specific phenotypes. However, this demonstrates the potential for ushering in an era of biological design, where humans design the genomes and characteristics of living organisms.

## 5. Conclusions

Genomics has entered an era where AI models not only classify signals but also infer mechanisms and increasingly generate biological sequences. Classical ML provided the first scalable tools for genotype–phenotype mapping; deep learning then expanded the solution space, with CNNs uncovering local sequence rules, RNN/LSTMs capturing positional and temporal dependencies, and transformers learning distal regulatory grammar across hundreds of kilobases. Foundation models such as DNABERT, Enformer, and Nucleotide Transformer demonstrate that pretraining on unlabeled sequence augmented by multi-omics yields transferable representations for promoters, TF binding, splicing, methylation, and variant-effect prediction. These advances in AI tools have substantially propelled genomics, enabling the capture of features that were difficult to infer using traditional methods and tools. Moreover, the progress of generative models suggests that in the near future, we stand at the threshold of moving beyond genome analysis to genome design, enabling controllable sequence design under appropriate safeguards. At present, generative models such as EVO can replicate or design CRISPR guide sequences and functional gene sequences, and GANs are being used to prototype human artificial genomes. In the near future, algorithms may emerge that construct whole, functional genomes conditioned on phenotype or predict phenotypes directly from genotype.

## Figures and Tables

**Table 1:** Early machine learning algorithms and application of genomic research.

Algorithm	Method	Application	References
Logistic regression	estimates the probability	Detection of SNP-SNP/gene interaction; Breeding and selection	[29,30]
Random Forest	Generate random classification and regression trees	Disease-associated SNP detection (Cancer, Alzheimer’s disease)	[31,32]
k-nearest neighbor	Utilize the k closest training instances in the feature space	SNP-SNP interaction detecting Microarray data analysis	[33,34,35]
Boosting machine	regression and classification that iteratively reduce residual	SNP pattern analysis for disease prediction Disease diagnosis (Cancer, Alzheimer’s disease)	[23,36] [37,38,39]
Naïve bayse	Bayes’ theorem to relate prior and posterior probabilities	Select biomarker SNP analysis of disease diagnosis (Alzheimer’s disease)	[27,40]
Supporting vector machine	Hyperplane that maximizes the margin between classes	Disease diagnosis Predict cancer-associated genes	[28,41,42]

**Table 2:** Methods and features of deep learning architectures used in genomic research.

Model Architecture	Method	Strengths	AI Model of Applied Genomics
DNN/MLP	Fully connected layer structure	Simple and fast to implement Applicable to small datasets	EP-DNN
CNN	Convolutional, pooling, and fully connected layer structure	Learns local sequence patterns Efficient resource utilization through weight sharing Fast training	DeepBind DeeperBind Basset DanQ
RNN/LSTM	Recurrent layer structure	Strong in position dependence Variable-length input possible	AttentiveChrome SG-LSTM-FRAME
Transformer	Self-attention mechanism	Capturing long-range interactions; Parallel processing Easily extended to pretraining	Enformer DNABERT Nucleotide Transformer
Hyena	long-context convolutional sequence model	Handles long sequences Maintains single-base resolution High performance with fewer parameters and memory efficient	HyenaDNA EVO

**Table 3:** Architectures and characteristics of major deep learning models proposed and applied to genomics research.

Function	Model	Architecture	Characteristics	Advantages	References
DNA/RNA binding protein binding sequence prediction	DeepBind	CNN	Protein–DNA/RNA binding prediction	- The first CNN-based protein binding predictor - simple to train	[53,64]
	iDeepS	CNN+LSTM	RNA–protein binding site prediction	- simple hybrid structure	[74]
	DanQ	CNN+LSTM	Regulatory feature prediction	- Strong performance on chromatin feature prediction	[65]
	TransBind	Transformer	Regulatory feature prediction	- Higher accuracy than CNN and RNN models	[75]
Epigenomic features	DeepChrome	CNN	Gene expression prediction from histone-modification profiles	- First CNN linking histone marks to gene activity - Simple and effective	[77]
	AttentiveChrome	LSTM+Attention	Gene expression prediction with attention over histone marks	- LSTM and attention improve interpretability - Identifies key histone marks driving expression	[68]
	CpGenie	CNN	DNA methylation prediction	- Strong performance on bulk methylation data	[79]
	DeepCpG	CNN	DNA methylation prediction	- Effective for single-cell methylation	[78]
	Enformer	Transformer	Gene expression, chromatin profile prediction	- Transformer captures long-range dependencies - Enables variant effect prediction	[19]
	EpiGePT	Transformer	Mapping gene expression levels from epigenetic marks	- Enables variant effect prediction - Outperforms CNN-based models	[81]
Splicing	DeepSplice	CNN	Alternative splicing detection	- Easy to train	[84]
	SpliceRover	CNN	Splice site detection	- High accuracy CNN for splice junctions	[85]
	SpliceAI	CNN	Quickly rank splicing effects by sequence	-Direct learning of long-distance context	[86]
miRNA	miRe2e	Transformer	Pre-miRNA prediction (Sequence, structure, MFE)	- Specialized for pre-miRNA prediction	[90]
	SG-LSTM-FRAME	LSTM	miRNA–mRNA interaction prediction	- High accuracy in miRNA target prediction	[92]
Non-canonical DNA structure	DeepZ	CNN+RNN	Z-DNA forming potential	- Specialized for left-handed Z-DNA detection	[67]
Genome function from sequence	DANN	DNN	Using a similar set of CADDs, but with weights automatically learned by deep learning.	- Wide application - Higher accuracy than existing machine learning	[96]
	BPNet	CNN	Base-resolution chromatin profiling	- Base-level motif discovery - Biologically validated	[73]
	DNABERT	Transformer	k-mer BERT pretraining and fine-tuning for tasks	- Flexible for multiple downstream tasks such as promoter, enhancer, and splicing site	[54]
	Borzoi	Transformer	Regulatory prediction, Using Enformer architecture	- Successor to Enformer with improved accuracy - Better representation of distal interactions	[80]
	HyenaDNA	Hyena	Long-range sequence prediction	- Efficiently models 10~35kb genomic contexts	[97]
	Nucleotide Transformer	Transformer	Masked sequence prediction	- Learns rich sequence representations without labels	[98]

## Data Availability

No new data were created or analyzed in this study. Data sharing is not applicable to this article.
